# Effect of tiomolibdate choline on copper balance in patients with Wilson disease: An open-label phase 2 trial

**DOI:** 10.1097/HC9.0000000000000971

**Published:** 2026-05-19

**Authors:** Aftab Ala, Thomas D. Sandahl, Karl H. Weiss, Valentina Medici, Chandler D. Robinson, Andrew Cittadine, Declan Tuffy, Edward Gane, Fred K. Askari

**Affiliations:** 1 The Roger Williams Institute of Liver Studies, Faculty of Life Sciences and Medicine, King’s College London, King’s College Hospital NHS Foundation Trust, London, UK; 2 Department of Hepatology and Gastroenterology, Aarhus University Hospital, Aarhus, Denmark; 3 Department of Internal Medicine, Salem Medical Center, Heidelberg, Germany; 4 Division of Gastroenterology and Hepatology, Department of Internal Medicine, University of California, Davis, California, USA; 5 Monopar Therapeutics, Wilmette, Illinois, USA; 6 Medical and Health Sciences, University of Auckland, Auckland, New Zealand; 7 Division of Gastroenterology, University of Michigan, Ann Arbor, Michigan, USA

**Keywords:** chelation therapy, copper, drugs, liver disease, neurology

## Abstract

**Background:**

Wilson disease (WD) is a rare disorder of copper (Cu) disposition. Tiomolibdate choline (TMC, bis-choline tetrathiomolybdate, ALXN1840), an oral Cu-binding agent, was investigated for the treatment of WD. The effect of repeat-dose TMC on Cu balance, serum Cu parameters, and safety was evaluated in an open-label, single-arm phase 2 study in 9 patients with WD.

**Methods:**

Enrolled patients with WD were admitted to a clinical research unit (CRU) and initiated on a Cu-controlled diet. Copper intake (food and drink) and output (feces and urine) were collected during a baseline period (days −4 through −1) and after initiation of daily TMC (days 1–8 and 25–39). Patients were allowed to leave the CRU during an interim period (days 9–24) but continued outpatient TMC treatment. Patients started on 15 mg/day TMC on day 1 with planned dose escalation to 30 mg/day TMC on day 29.

**Results:**

TMC showed a statistically significant reduction in daily Cu balance from baseline over the entire study period (days 1–39), attributed to increased fecal Cu excretion. The decrease in Cu balance was rapid, with a statistically significant decrease beginning on day 4. The cumulative mean decrease from baseline in Cu balance was −6.08 mg over 21 days. Plasma total Cu and directly measured non-ceruloplasmin-bound Cu levels increased immediately, consistent with Cu mobilization into blood and formation of stable tripartite complexes of Cu, TMC, and albumin. TMC was well tolerated. Alanine aminotransferase elevations that occurred were mild and reversible with dose modulation.

**Conclusion:**

TMC’s reduction in Cu balance in WD patients, along with strong mobilization of Cu, supports the therapeutic potential of TMC in WD.

## Graphical abstract

**Figure:**
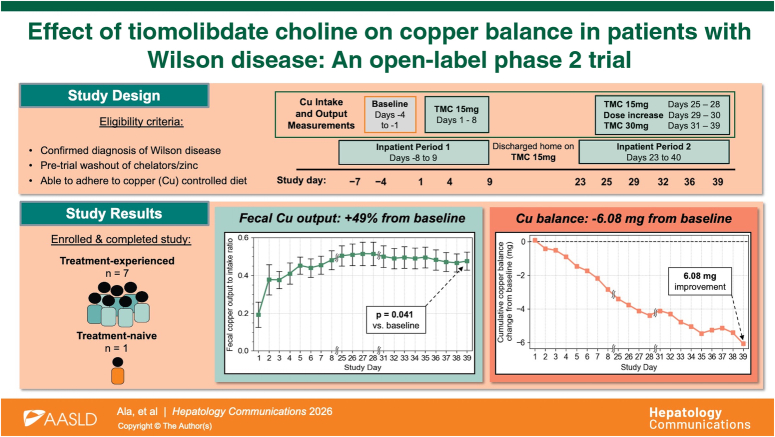

## INTRODUCTION

Wilson disease (WD) is a rare, inherited, autosomal recessive disorder with hepatic, psychiatric, and neurologic damage resulting from excess hepatic copper (Cu) accumulation due to mutations in the P-type ATPase Cu transporter ATP7B.^1^^–^^3^ Excess hepatic Cu enters circulation as exchangeable Cu loosely bound with albumin and deposits in extrahepatic organs, especially the brain.^1^^,^^4^ Urinary Cu excretion substantially increases in untreated WD but cannot compensate for the Cu overload in the liver.^2^

The standard of care (SoC) in WD is lifelong therapy with oral chelating agents, such as D-penicillamine (DPA) and trientine, or zinc salts.^4^ DPA and trientine bind Cu in blood and promote urinary Cu excretion, the magnitude of which is greatest during initial treatment but declines over time.^5^^–^^7^ Zinc induces metallothionein expression in enterocytes, which inhibits intestinal absorption of dietary and endogenously secreted Cu. Copper–metallothionein complexes are excreted in feces.^1^^,^^2^^,^^4^ The current oral chelators can be associated with serious adverse events, including paradoxical neurologic worsening, bone marrow toxicity, and skin changes, necessitating switching to alternative treatments.^4^ Inconvenient multi-dose-per-day regimens away from food and other medications, along with dietary restrictions, contribute to nonadherence.^1^^,^^8^ Approximately one quarter of patients with neurological WD paradoxically worsen after starting chelator therapy, and in some cases, this worsening can be irreversible.^9^^,^^10^

Tiomolibdate choline (TMC; Monopar Therapeutics, Wilmette, IL) is a once-daily oral Cu–protein-binding molecule in development for the treatment of WD.^11^ Preclinical and clinical studies show TMC can bind, mobilize, and sequester Cu.^12^^–^^14^ Its active moiety, the tetrathiomolybdate (TTM) anion, selectively binds Cu with high affinity and functions as an albumin tripartite complex activator, forming a stable TMC–albumin–Cu complex.^15^^,^^16^ In previous studies, sequestration of toxic Cu in tripartite complexes (TPCs) was thought to be the primary driver of the neurologic improvement observed in WD patients treated with TTM.^17^^,^^18^ In animals, TMC significantly reduced liver Cu concentrations and promoted biliary excretion of Cu.^12^^,^^19^ In humans, TMC administration significantly reduced intestinal absorption of Cu and accumulation of Cu in the liver and brain.^20^ In phase 2 and phase 3 studies, TMC treatment resulted in greater mobilization of Cu compared with the current standard of care, measured as the area under the effect curve for directly measured non-ceruloplasmin-bound Cu (dNCC).^21^ More importantly, sustained and significant improvements in neurologic symptoms and hepatic status were observed in WD patients treated with TMC through 6 years.^22^ TMC was well tolerated across clinical studies.

The present study was conducted to evaluate the effect of repeat-dose TMC on Cu balance in WD patients. A recently published peer-reviewed letter to the editor highlighted the importance of comparing to the pre-treatment baseline when assessing the effect of a potential WD treatment on Cu balance.^23^ We report herein the Cu balance change from baseline results from an open-label, single-arm phase 2 study of WD patients on TMC treatment. The objective of this study was to evaluate the effects of repeat-dose TMC on Cu balance, Cu mobilization, and safety in patients with WD.

## METHODS

The protocol, amendments, and other relevant study documents were approved by an Independent Ethics Committee (London Bridge Research Ethics Committee, London, UK). The trial was conducted according to the ethical principles of Good Clinical Practice, the Declaration of Helsinki, and the International Council for Harmonization of Technical Requirements for Pharmaceuticals for Human Use Harmonized Tripartite Guideline. Written informed consent was obtained from all participants.

### Study design

**FIGURE 1:**
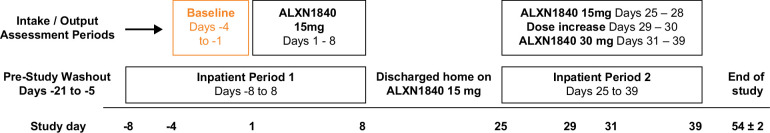
Study schematic.

This open-label, single-arm phase 2 trial evaluated the effect of repeat-dose TMC (ALXN1840 tablets) on Cu balance, serum Cu parameters, safety, and tolerability in patients with WD. The trial was conducted at 2 centers in the United Kingdom and New Zealand between September 2020 and May 2022 and evaluated repeat-dose TMC administration for 39 days. The study schematic is provided in Figure 1.

Consented patients who met all inclusion criteria and none of the exclusion criteria were admitted to the clinical research unit (CRU) on study day −8. Decoppering therapies were discontinued from day −4 (DPA/trientine) or day −21 (zinc) to enable a baseline assessment of Cu balance in the absence of treatment. A Cu-controlled diet was initiated upon admission to the CRU to support dietary and gastrointestinal equilibration and continued throughout both inpatient periods (days −8 through 8 and days 25 through 39). Baseline measurements were obtained on days −4 through −1. Between days 9 and 24, participants could leave the CRU and continue dosing as an outpatient. Participants were encouraged to adhere to their usual Cu-controlled diet when not at the CRU. Participants received TMC 15 mg/day on days 1–28, followed by a planned dose increase to 30 mg/day on day 29.

### Study participants

Adult (age ≥18) patients with a confirmed diagnosis of WD by a score of ≥4 on the Leipzig diagnostic criteria (per 2012 European Association for the Study of the Liver WD Clinical Practice Guidelines) or by historic test results were eligible for enrollment.^1^^,^^24^ Eligible patients agreed to adhere to dietary and contraception requirements and lifestyle restrictions.

Patients with clinically significant current or past medical history (eg, chronic constipation, irritable bowel syndrome, decompensated cirrhosis, elevated ALT levels, and advanced renal or neurologic disease) were excluded. Patients were also excluded if they had a history of illicit drug abuse or a history of significant alcohol abuse within 1 year before screening. Alcohol consumption was prohibited within 48 hours before the study.

Participants were considered treatment-experienced if they had received prior treatment with DPA, trientine, or zinc for ≥28 days, while those who received prior treatment for <28 days were considered treatment-naïve.

### Treatment

During the first treatment period (days 1–28), participants received TMC 15 mg/day, which was planned to be increased to 30 mg/day on day 29. Before dose escalation, the Safety Review Committee reviewed available safety data through day 23 for each participant, and those with adverse safety signals were reduced to 15 mg every other day.

In the CRU, TMC was administered orally after an overnight fast (ie, ≥10 h) at the same time every morning under direct medical supervision. Meals were scheduled ≥2 hours after dosing. When participants were not in the CRU, they were instructed to take TMC at approximately the same time each day (±1 h); meals were to be delayed to ≥2 hours after dosing. Participants used short message service text messaging to confirm TMC administration during the outpatient period.

### Outcomes

Efficacy endpoints included mean daily Cu balance, change from baseline in mean daily Cu balance, and the proportion of Cu recovered in output (feces, urine, and total) relative to Cu intake (food + drink). Serum Cu parameters assessed included total plasma Cu, dNCC, and ceruloplasmin-bound Cu (Cp-Cu).

### Assessments

#### Copper balance

Cu balance was defined as the difference between total intake (from food and drink) and output (from urine and feces) of Cu. Participants were initiated on a standardized Cu-controlled diet with a limited meal selection. Total input was quantified by monitoring and recording individual participants’ intake, including food and liquids.

Copper output was quantitatively assessed by monitoring and recording participants’ bowel movements and urination. Urine samples were pooled daily, and the volume was recorded during a 24-hour period. Fecal samples were individually collected and weighed. Copper concentrations from samples were determined by inductively coupled plasma mass spectroscopy (ICP-MS). Total Cu intake and output were assessed beginning on day −4 before initiation of TMC (baseline, day −4 to day −1) and during both inpatient periods (days 1–8 and 25–39). Total Cu intake and output were not assessed during the outpatient period (days 9–24). Treatment periods were defined as the 15 mg/day treatment period (days 1–8 and 25–28, “days 1–28”) and the overall study period (days 1–8, 25–28, and 31–39, “days 1–39”). Change from baseline in Cu balance was defined as the difference between the average daily Cu balance for the baseline period and the average daily Cu balance for the period of interest.

#### Safety

Safety assessments included physical examinations, recording of vital signs, and electrocardiograms (ECG), and clinical laboratory tests. Adverse events reported by participants were collected and coded using the Medical Dictionary for Regulatory Activities (MedDRA) v22.0 or higher.

#### Serum Cu parameters

Plasma total Cu, dNCC, and Cp-Cu were measured following initiation of TMC. Plasma total Cu encompassed all Cu species, including ceruloplasmin-bound (Cp-Cu) and non-ceruloplasmin-bound (dNCC). To speciate Cu, a highly specific anticeruloplasmin antibody was used to remove Cp-Cu from plasma total Cu. The supernatant (dNCC) contained Cu not bound with ceruloplasmin, including TMC–albumin–Cu TPCs. Copper content of all PD parameters was quantified by ICP-MS.^25^

### Statistical analysis

A sample size of ~10 participants was based on the feasibility of recruitment, given the rarity of WD in the United Kingdom and New Zealand, and the required voluntary CRU confinement. All efficacy analyses were performed on completers only, defined as participants who met all eligibility criteria and remained enrolled through day 39 (end of study), which was all participants but one (who failed to discontinue DPA during the run-in period). All safety analyses were performed on the full analysis set, defined as all participants who received ≥1 dose of TMC and met all inclusion criteria and none of the exclusion criteria. Serum Cu parameter analyses were performed on all participants with sufficient samples for calculating the serum Cu parameters.

In the case of missing stool samples or stool irregularity, Cu stool output was averaged over the days between bowel movements (as needed) to approximate values for each 24-hour period.

Continuous data were summarized using descriptive statistics, and categorical variables were presented as frequencies and percentages. *p*-values were calculated using 2-tailed paired *t* tests. Analyses were performed using SAS software (SAS Institute, Inc., Cary, NC, USA), version 9.4 or higher, and Python (Python Software Foundation, Beaverton, OR, USA), version 3.13 or higher.

## RESULTS

### Participant baseline characteristics

Nine participants with WD were enrolled, 8 treatment-experienced (cohort 1) and 1 treatment-naïve (cohort 2). Participant baseline characteristics are summarized in Table 1. Most participants were male (77.8%) and White (88.9%), with a mean (SD) age of 34.1 (12.0) years. The median time since diagnosis of WD was 147.6 months (range, 20.8–563.0 months) for the treatment-experienced cohort. The treatment-naïve participant was diagnosed with WD 3 months before the study and had not yet started treatment.

**TABLE 1:** Baseline characteristics of all enrolled participants

Characteristic	Cohort 1, n=8	Cohort 2, n=1	Total, n=9
Age (years), mean (SD)	35.1 (12.41)	26.0 (NC)	34.1 (12.0)
Sex, n (%)			
Male	7 (87.5)	0	7 (77.8)
Female	1 (12.5)	1 (100)	2 (22.2)
White race, n (%)	7 (87.5)	1 (100)	8 (88.9)
Body mass index (kg/m^2^), mean (SD)	30.2 (7.19)	19.4 (NC)	29.0 (7.63)
Time since WD diagnosis (months), mean (SD)	203.9 (195.11)	3.1 (NC)	181.6 (194.40)
Cirrhosis (yes), n (%)	3 (37.5)	0	3 (33.3)
Cumulative duration of prior WD treatment (months), mean (SD)	192.3 (176.48)	NA	192.3 (176.48)
Prior WD therapy		NA	
Penicillamine or trientine (±zinc)	7 (87.5)		7 (77.8)
Penicillamine (±zinc)	5 (62.5)		5 (55.6)
Penicillamine and trientine (±zinc)	1 (12.5)		1 (11.1)
Trientine (±zinc)	1 (12.5)		1 (11.1)
Zinc monotherapy	1 (12.5)		1 (11.1)

Abbreviations: NA, not applicable; NC, not calculated; SD, standard deviation; WD, Wilson disease.

One participant from cohort 1 discontinued participation on day 4 due to a major protocol deviation (failure to discontinue DPA during the run-in period); all other participants completed the study. All participants were 100% compliant with the TTM dosing regimen. Five participants had a dose increase to 30 mg/day as planned beginning day 29, and 3 participants had dose reductions to 15 mg every other day, due to grade 1 or 2 elevated ALT values.

### Copper intake and output

**FIGURE 2:**
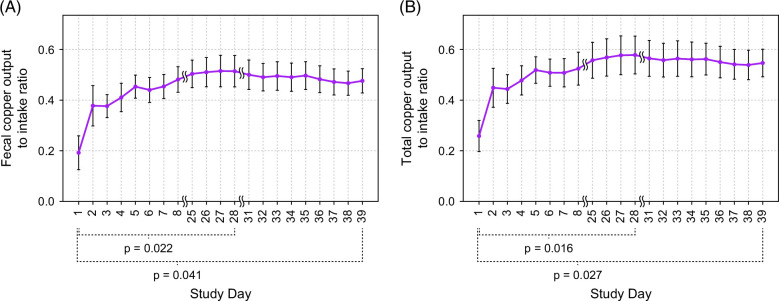
Mean daily copper output-to-intake ratio, rolling average (n=8). (A) Daily fecal copper output-to-intake ratio. (B) Total copper output-to-intake ratio. Rolling averages are calculated as the average of a patient’s daily values from day 1 through day X, where X ranges from 1 through 39. Standard error of the mean.

The majority (>99%) of dietary Cu intake was from food. Mean (SD) daily Cu intake during the overall study period was 1.73 (0.19) mg (Table 2). Copper output was driven primarily by fecal excretion. The mean (SD) daily fecal Cu output increased from 0.54 (0.28) mg at baseline to 0.81 (0.22) mg during the 15 mg/day treatment period and 0.78 (0.21) mg during the overall study period (Table 2). To control for day-to-day variability in Cu intake when assessing daily Cu output, fecal Cu output as a ratio of daily Cu intake was calculated. The mean (SD) daily fecal Cu output-to-Cu intake ratio increased by 49% from 0.31 (0.17) at baseline to 0.48 (0.14) during the whole study period (*p*=0.041; Figure 2A). As anticipated, only low amounts of Cu were excreted in the urine (Table 2). The mean (SD) daily total copper output-to-intake ratio increased by 43% from 0.39 (0.19) at baseline to 0.55 (0.15) by day 39 (*p*=0.027; Figure 2B).

**TABLE 2:** Copper intake and output by period

	Total (n=9)^a^		
	Baseline	Days 1–28	Days 1–39
Copper input (mg)			
Total	1.82 (0.30)	1.67 (0.23)	1.73 (0.19)
Copper output (mg)			
Total	0.71 (0.34)	0.92 (0.28)	0.90 (0.25)
Feces	0.54 (0.28)	0.81 (0.22)	0.78 (0.21)
Urine	0.19 (0.20)	0.13 (0.07)	0.15 (0.06)
Output-to-intake ratios			
Feces-to-intake	0.31 (0.17)	0.51 (0.18)	0.48 (0.14)
Urine-to-intake	0.10 (0.09)	0.09 (0.05)	0.09 (0.04)
Total output-to-intake	0.39 (0.19)	0.58 (0.21)	0.55 (0.15)

*Note:* All data presented as mean (standard deviation) unless otherwise noted.

Baseline consists of days −4 through −1; days 1–28 consist of days 1–8 and 25–28; days 1–39 consist of days 1–8, 25–28, and 31–39.

^a^ One participant who failed to discontinue pre-study D-penicillamine was excluded from analyses after baseline.

### Change in Cu balance from baseline

**FIGURE 3:**
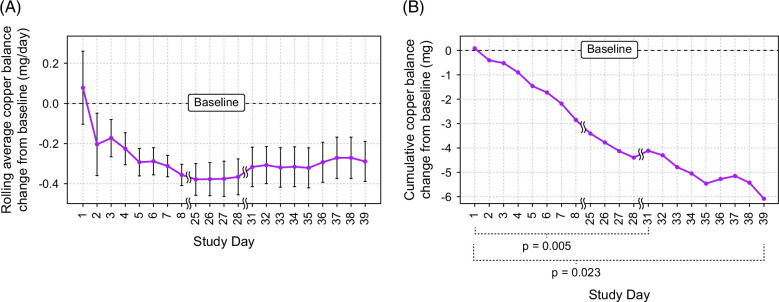
Mean daily copper balance change from baseline (n=8). (A) Rolling average with standard error about the mean. (B) Cumulative sum. Rolling average calculated as the average of a patient’s daily copper balance change from baseline from day 1 through day X, where X ranges from 1 through 39. Cumulative sum calculated as the sum of a patient’s daily copper balance change from baseline from day 1 through day X, averaged over X days, where X ranges from 1 through 39.

The mean daily Cu balance reduced from baseline beginning on day 2 and remained decreased across the whole study period (Figure 3A). The mean (SD) daily Cu balance change from baseline for the 15 mg/day treatment period (days 1–28) was −0.41 (0.24) mg (*p*=0.004) for cohort 1 and −0.37 (0.25) mg (*p*=0.005) for all participants. The mean (SD) daily Cu balance change from baseline for the overall study period (days 1–39) was −0.33 (0.28) mg (*p*=0.019) for cohort 1 and −0.29 (0.28) mg (*p*=0.023) for all participants (Table 3). Over the 21 days for which data were collected, the mean cumulative decrease from baseline in Cu balance for all participants was −6.08 mg [95% confidence interval (CI), −10.18 to −1.98; Figure 3B].

**TABLE 3:** Daily net copper balance

	Baseline	Days 1–28	Days 1–39
Daily copper balance in mg, mean (SD)			
** **Cohort 1 (n=8)^a^	1.18 (0.27)	0.77 (0.38)	0.85 (0.28)
** **Change from baseline		−0.41 (0.24)	−0.33 (0.28)
** **Cohort 2 (n=1)	0.51 (NA)	0.44 (NA)	0.52 (NA)
** **Change from baseline		−0.07 (NA)	0.005 (NA)
** **Total (n=9)^a^	1.11 (0.33)	0.73 (0.37)	0.81 (0.29)
** **Change from baseline		−0.37 (0.25)	−0.29 (0.28)

*Note:* Baseline consists of days −4 through −1; days 1–28 consist of days 1–8 and 25–28; days 1–39 consist of days 1–8, 25–28, and 31–39.

^a^ One participant who failed to discontinue pre-study D-penicillamine was excluded from analyses after baseline.

Abbreviations: NA, not applicable; SD, standard deviation

### Serum Cu parameters

**FIGURE 4:**
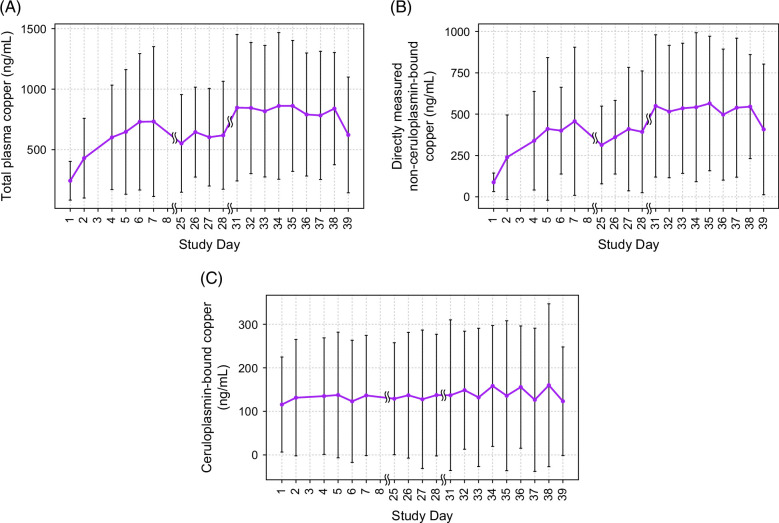
Mean daily serum copper parameters (n=8). (A) Mean daily total plasma copper. (B) Mean daily directly measured non-ceruloplasmin-bound copper (dNCC). (C) Mean daily ceruloplasmin-bound copper. All values presented as ng/mL. Standard deviation about the mean.

Following repeated oral dose administration of TMC, plasma concentrations of total Cu and dNCC increased immediately, and trough concentrations were reached between days 4 and 7. Mean daily total plasma Cu concentrations increased steadily between days 1 and 7 (Figure 4A). Treatment with TMC mobilized Cu and increased plasma concentration of dNCC (Figure 4B). Ceruloplasmin-bound Cu concentrations remained stable throughout the treatment period and were unchanged through day 39 from baseline concentrations on day 1 (Figure 4C).

### Safety

Administration of TMC was generally well tolerated with a safety profile consistent with other TMC studies. A total of 22 treatment-emergent adverse events (TEAEs) were reported in 7/9 participants (77.8%); no serious adverse events or deaths were reported (Table 4). Of the 22 TEAEs reported, 20 (90.9%) were mild, and no event led to TMC discontinuation. Two participants (22.2%) experienced hepatic TEAEs (abnormal liver function test, transaminase increased) that resulted in dose reductions; both events were deemed treatment-related and resolved without additional intervention. No other clinically significant changes were observed in clinical laboratory values, vital signs, physical examinations, or ECG results.

**TABLE 4:** Overview of treatment-emergent adverse events

	Safety set (n=9)
	n (%)
Any AE	7 (77.8)
** **Related	2 (22.2)
** **Not related	7 (77.8)
Any SAE	0
AE by toxicity	
** **Grade 1	6 (66.7)
** **Grade 2	2 (22.2)
** **Grade 3	0
** **Grade 4	0
** **Grade 5	0
AE leading to withdrawal of the study intervention	0

Abbreviations: AE, adverse event; SAE, serious adverse event.

## DISCUSSION

Repeat oral dosing of TMC resulted in a rapid, statistically significant reduction from baseline in the average daily Cu balance. This was driven primarily by an increase of over 40% in fecal Cu output induced by TMC. When accounting for dietary variability by analyzing fecal Cu output as a proportion of Cu intake, the increase from baseline was even greater, at ~50%. This effect was observed to begin on day 2 following treatment initiation and gradually increased, reaching its maximal effect by day 5 and persisting for the remainder of the study. In this study, TMC treatment resulted in a −6.08 mg cumulative decrease from baseline in Cu balance (−0.29 mg/day reduction), translating to −105.7 mg in a year if the Cu balance were extrapolated as steady state. When looking at only the 15 mg/day treatment period of the study, the cumulative reduction was even greater, −0.37 mg/day (translating to −133.8 mg per year if the Cu balance were extrapolated as a steady state).^23^ As the pathology of WD is driven by progressive Cu accumulation, the improved Cu balance resulting from treatment with TMC may limit disease progression and yield long-term symptomatic improvements. In a pooled analysis of 255 WD patients treated with TMC in phase 2 and 3 clinical trials, statistically significant and persistent improvements in neurological outcomes, as measured by the Unified Wilson Disease Rating Scale (UWDRS) and the Clinical Global Impressions (CGI) scale, were observed over 6 years of treatment.^22^ Hepatic improvement was also demonstrated by a decrease from baseline through week 312 in the New Wilson Index (NWI) score, a prognostic scoring system comprising assessments of liver damage and systemic inflammation.^26^^,^^27^

Our study demonstrates TMC’s ability to remove Cu and improve Cu balance even in WD patients extensively treated with SoC. Statistically significant increases in fecal Cu, dNCC, and total plasma Cu were seen with TMC treatment in patients with a mean prior WD treatment duration of over 16 years, suggesting that current SoC leaves a considerable amount of residual Cu in the body. This finding is further supported by the results from a completed 48-week phase 3 randomized, controlled trial of TMC in WD patients that successfully met its primary endpoint and showed Cu mobilization even in patients randomized to TMC who had a mean prior SoC therapy duration of 11.2 years.^28^ Mobilization of Cu with TMC in the setting of years-long oral chelator therapy is likely a result of the much greater Cu-binding affinity of TMC compared with DPA and trientine.^29^

Increased fecal Cu excretion was observed with TMC treatment when analyzed as both an absolute amount and as a ratio to control for variability in day-to-day Cu intake. Preclinical studies have reported the ability of Cu and molybdenum to be excreted into the bile following administration of TTM in animal models of WD. In 2 separate studies, intravenous (i.v.) administration of 10 mg/kg ammonium TTM in a WD rat model resulted in biliary excretion of Cu and molybdenum in a near 1:1 molar ratio over the next 4–12 hours.^30^^,^^31^ The required fasting period in our study of 10 hours before through 2 hours after TMC administration likely underestimates the potency of dietary Cu blockade and suggests that a meaningful amount of Cu measured in fecal output could be systemic in origin.

Cu balance studies are challenging to conduct. A thorough and complete collection is difficult, and it is not feasible to fully capture and measure all routes of Cu excretion beyond the typical feces and urine, such as hair loss, skin exfoliation, tears, and sweat. Enrollment in the study was challenging due to the rarity of WD as well as the feasibility of recruitment for a study that required voluntary confinement to a clinical research facility for multiple weeks. Recruitment of treatment-naïve patients with WD was limited by the low incidence of new diagnoses of WD in the geographic regions where the study was performed. These challenges are not unique to this study and likely contribute to the lack of Cu balance studies in recent years, with the most recent study in patients with WD having been published over 30 years ago.^14^ Because this study was designed as a short-term mechanistic evaluation of the effect of TMC on Cu elimination, clinical outcome data were not collected, and as a result, the relationship between increased fecal Cu excretion and WD outcomes such as neurologic improvement could not be assessed.

In summary, our results demonstrate that TMC treatment leads to a rapid improvement in daily Cu balance in WD patients, driven by a statistically significant increase in fecal Cu excretion. The improved Cu balance was sustained throughout the duration of the study. This sustained improvement, along with sequestration of toxic Cu in the bloodstream by TPC formation, may underlie the long-term benefit observed in multiple clinical studies of WD patients on TMC treatment. The results of this study support the therapeutic potential of TMC in WD.
